# Non‐Transfusion‐Dependent Thalassemia: An Image Gallery Worth a Thousand Words

**DOI:** 10.1002/ajh.27621

**Published:** 2025-01-30

**Authors:** Khaled M. Musallam, Sujit Sheth, Thomas D. Coates, Hanny Al‐Samkari, Maria Domenica Cappellini, Kevin H. M. Kuo, Vip Viprakasit, Ali T. Taher

**Affiliations:** ^1^ Center for Research on Rare Blood Disorders (CR‐RBD) Burjeel Medical City Abu Dhabi UAE; ^2^ Department of Public Health & Epidemiology Khalifa University Abu Dhabi UAE; ^3^ Division of Hematology/Oncology, Department of Pediatrics Weill Cornell Medicine New York New York USA; ^4^ Hematology Section, Cancer and Blood Disease Institute, Children's Hospital Los Angeles University of Southern California Los Angeles California USA; ^5^ Division of Hematology and Oncology, Massachusetts General Hospital Harvard Medical School Boston Massachusetts USA; ^6^ Unit of Medicine and Metabolic Disease Fondazione IRCCS Ca' Granda Ospedale Maggiore Policlinico Milan Italy; ^7^ Division of Hematology, Department of Medicine University of Toronto Toronto Ontario Canada; ^8^ Department of Pediatrics & Thalassemia Center, Faculty of Medicine Siriraj Hospital, Mahidol University Bangkok Thailand; ^9^ Department of Internal Medicine American University of Beirut Medical Center Beirut Lebanon

1

Our understanding of the molecular pathways and associated clinical presentations characterizing various thalassemia phenotypes has substantially improved over the years. Non‐transfusion‐dependent thalassemia (NTDT) refers to patients who present with mild–moderate anemia, which does not necessitate lifelong, regular transfusion therapy. This typically includes patients with β‐thalassemia intermedia, mild–moderate hemoglobin E/β‐thalassemia, and α‐thalassemia intermedia (hemoglobin H disease) (Figure 1) [1, 2, 3, 4, 5, 6, 7]. The underlying α/non‐α globin chain imbalance and subsequent ineffective erythropoiesis and peripheral hemolysis lead to chronic anemia, primary iron overload, and a hypercoagulable state. These, in turn, are associated with a range of clinical morbidities that can impact quality of life and lead to premature death (Figure 2) [2, 8, 9, 10, 11, 12, 13, 14, 15, 16, 17, 18, 19, 20, 21, 22]. In view of the growing evidence on the negative impact of untreated anemia in these patients, long‐term management becomes key. However, the only options that have been available so far include transfusions which can worsen iron overload and introduce transfusion‐dependence burden, off‐label use of hydroxyurea based on data from small trials or observational studies, and splenectomy which is associated with increased risks of infections and thrombosis (Figure 3) [6, 9, 23, 24]. Beyond anemia, cumulative iron overload due to increased intestinal iron absorption needs to be regularly monitored and managed with iron chelation therapy. Multimorbidity in NTDT also requires close monitoring and early intervention through a multidisciplinary team approach (Figure 4) [2, 6, 25, 26, 27]. In the last decade, we have witnessed several novel agents being developed to manage anemia in NTDT. Agents targeting hepcidin dysregulation have not been successful in clinical trials, despite encouraging data in animal models. Luspatercept, an erythroid maturation agent, showed efficacy in improving hemoglobin level in adults with β‐NTDT and is now approved in Europe (but not the United States). Mitapivat, a pyruvate kinase activator, has also shown efficacy in improving hemoglobin level and functional status in a recent phase 3 trials in adult patients with both α‐ and β‐NTDT (Figure 5) [24, 28, 29, 30]. Clinical management guidelines are now available, but awareness of the various morbidities and treatment options in NTDT, especially among patients remains essential (a patient friendly summary is provided in the Appendix S1).

**FIGURE 1:**
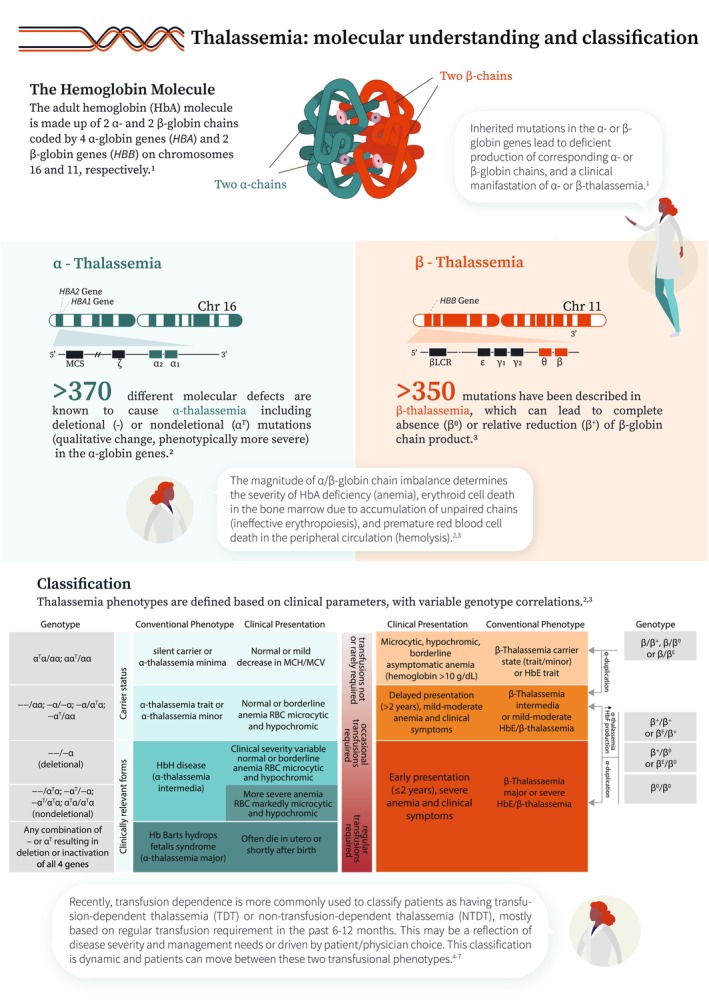
Thalassemia: Molecular understanding and classification [1, 2, 3, 4, 5, 6, 7].

**FIGURE 2:**
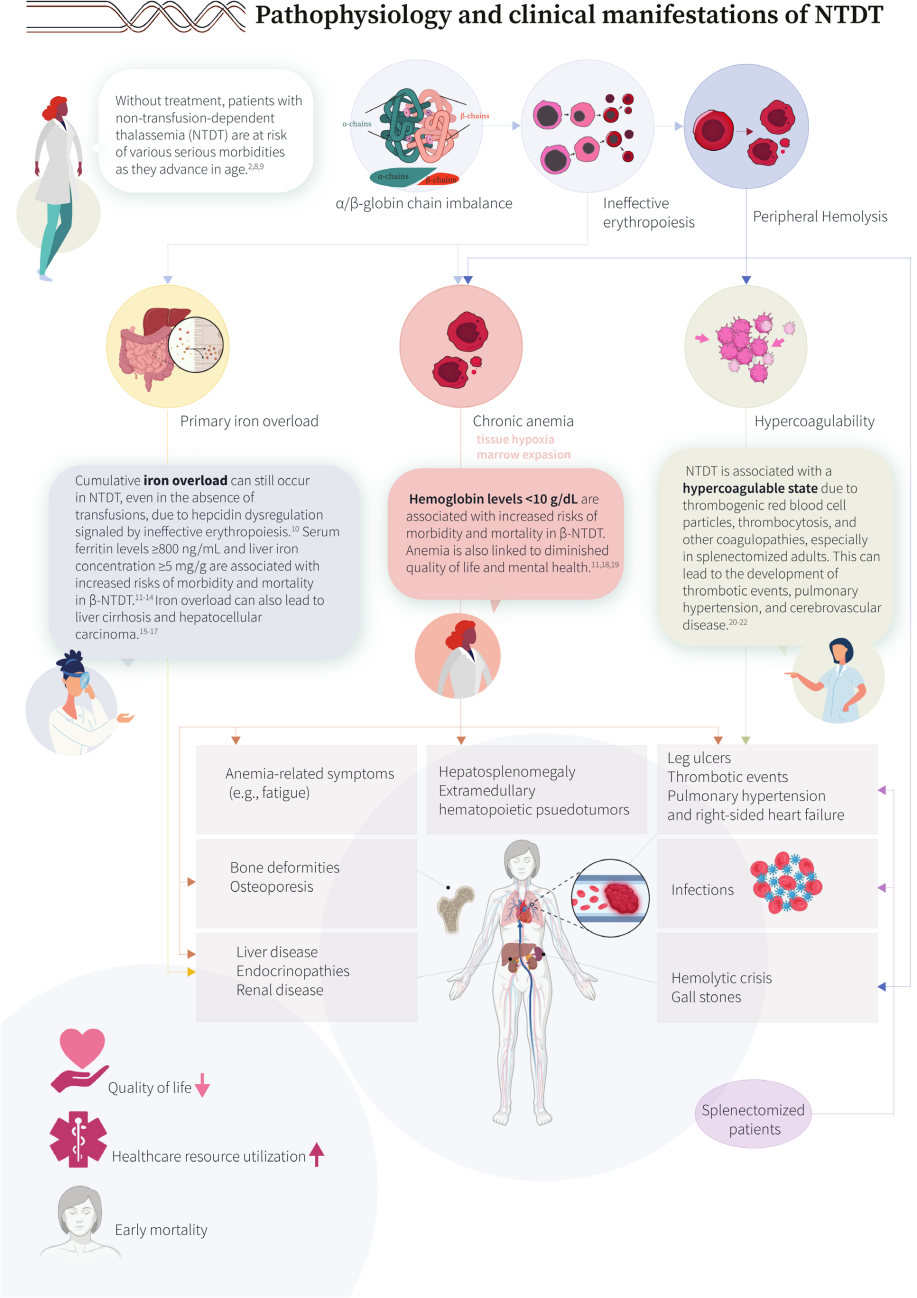
Pathophysiology and clinical manifestations of NTDT [2, 8, 9, 10, 11, 12, 13, 14, 15, 16, 17, 18, 19, 20, 21, 22].

**FIGURE 3:**
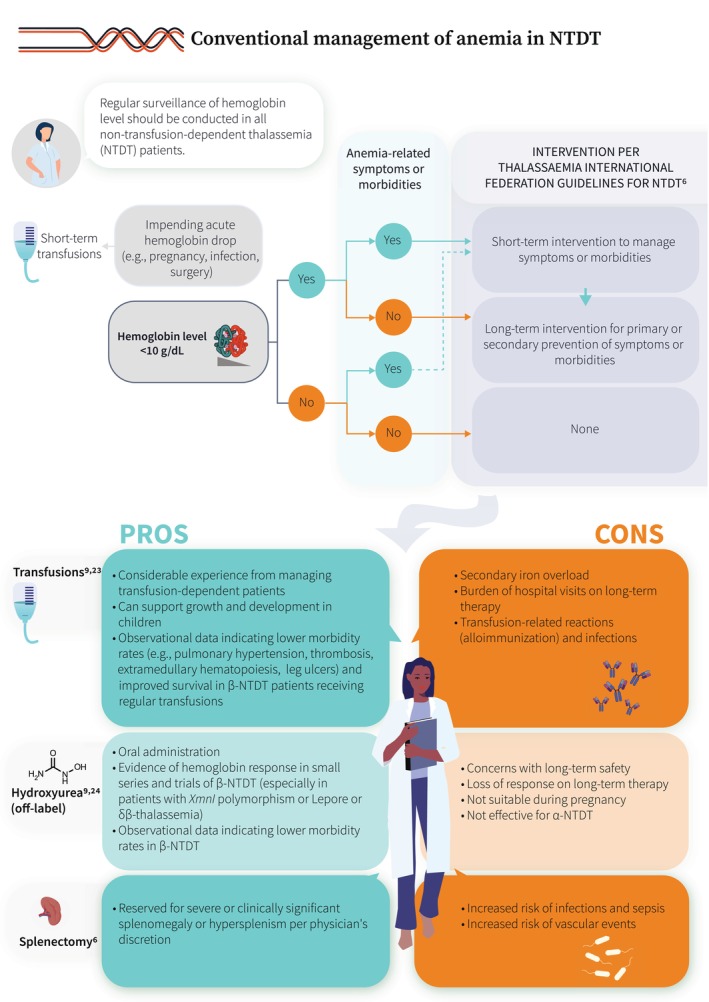
Conventional management of anemia in NTDT [6, 9, 23, 24].

**FIGURE 4:**
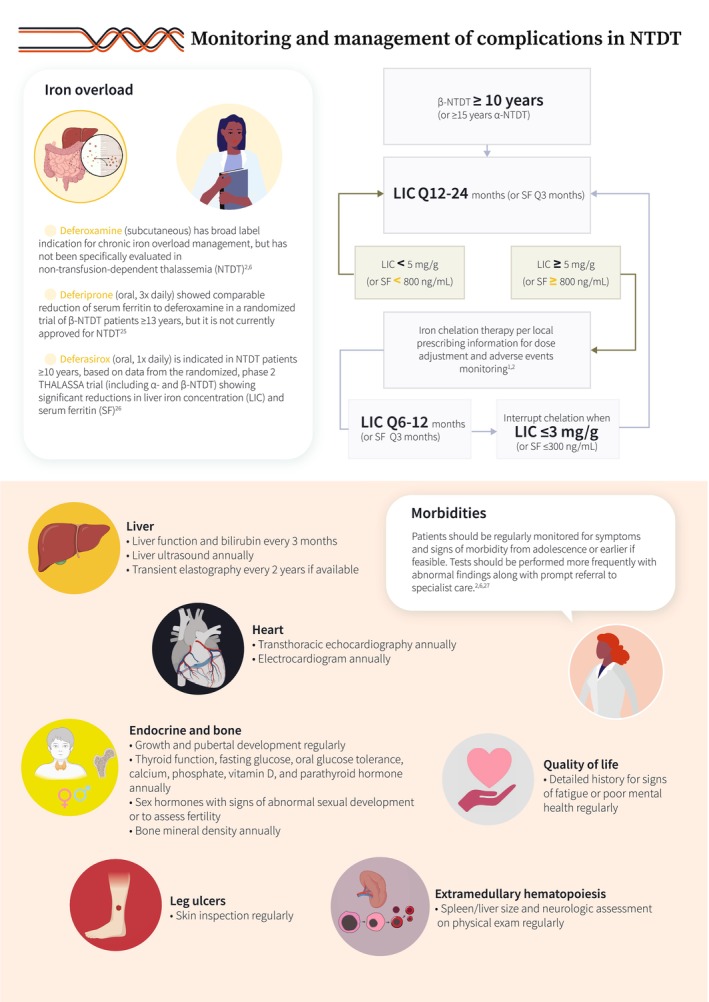
Monitoring and management of complications in NTDT [2, 6, 25, 26, 27].

**FIGURE 5:**
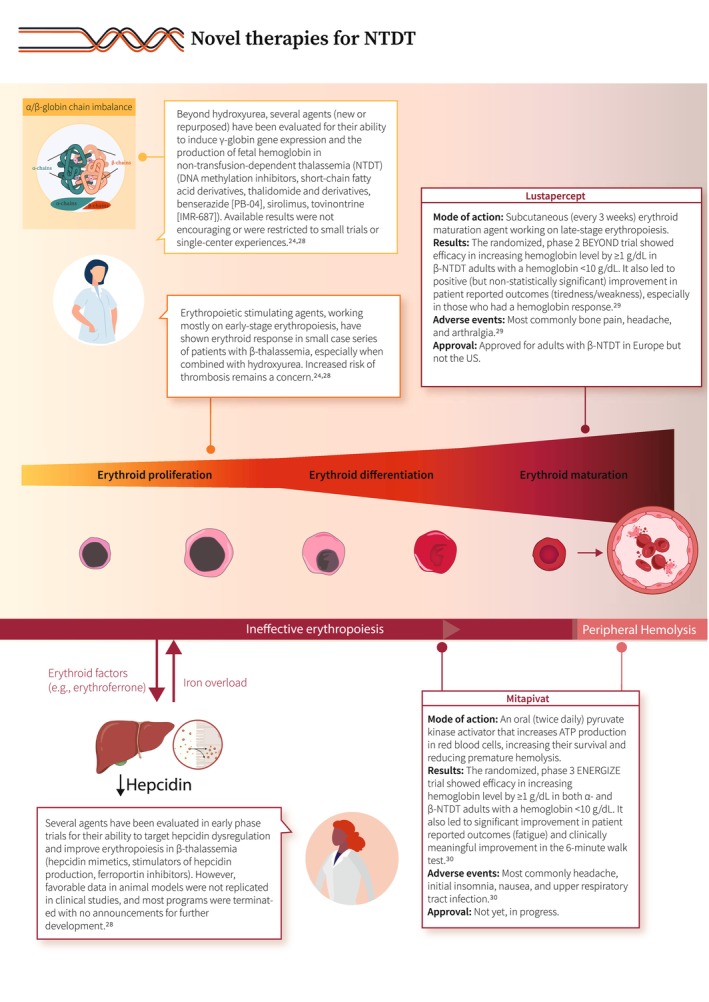
Novel therapies for NTDT [24, 28, 29, 30].

## Author Contributions

All authors contributed to conceptualization and manuscript drafting or critical review. K.M.M. was also involved in the creation of visualizations. All authors validated the manuscript and gave final approval for submission.

## Ethics Statement

Ethics approval not applicable as no patients were involved in this work.

## Conflicts of Interest

K.M.M. reports consultancy fees from Novartis, Bristol Myers Squibb (Celgene Corp), Agios Pharmaceuticals, CRISPR Therapeutics, Vifor Pharma, Novo Nordisk, and Pharmacosmos; and research funding from Agios Pharmaceuticals and Pharmacosmos. S.S. reports consultancy fees from Agios Pharmaceuticals, Bristol Myers Squibb, and Novo Nordisk; being a member of a clinical trial steering committee for Vertex Pharmaceuticals; and research funding (for clinical trials) from Agios Pharmaceuticals, Bristol Myers Squibb, Novo Nordisk, and Regeneron. T.D.C. reports advisory support to Agios Pharmaceuticals, Bristol Myers Squibb, and Chiesi. H.A.‐S. reports consultancy fees from Agios Pharmaceuticals, Alnylam, Alpine, Amgen, argenx, Novartis, Pharmacosmos, and Sobi; and research funding to institution from Agios Pharmaceuticals, Amgen, Novartis, Sobi, and Vaderis. M.D.C. reports consultancy fees from Novartis, Bristol Myers Squibb (Celgene Corp), Vifor Pharma, and Vertex Pharmaceuticals; and research funding from Novartis, Bristol Myers Squibb (Celgene Corp), La Jolla Pharmaceutical Company, Roche, Protagonist Therapeutics, and CRISPR Therapeutics. K.H.M.K. reports grants from Agios Pharmaceuticals and Pfizer; consulting fees from Agios Pharmaceuticals, Alexion Pharmaceuticals, Biossil, Bristol Myers Squibb, Forma, Novo Nordisk, Pfizer, and Vertex Therapeutics; honoraria from Agios Pharmaceuticals and Bristol Myers Squibb; and being on a data safety monitoring board/advisory board for Sangamo. V.V. reports grants from Agios Pharmaceuticals, Bristol Myers Squibb (Celgene Corp), DisperSol Technologies, IONIS Pharmaceuticals, Novartis, Pharmacosmos, The Government Pharmaceutical Organisation, and Vifor; and consulting fees from Agios Pharmaceuticals, Bristol Myers Squibb (Celgene Corp), DisperSol Technologies, IONIS Pharmaceuticals, Novartis, Pharmacosmos, and Vifor. A.T.T. reports consultancy fees from Novo Nordisk, Bristol Myers Squibb (Celgene Corp), Agios Pharmaceuticals, Pharmacosmos, and Roche; and research funding from Novo Nordisk, Bristol Myers Squibb (Celgene Corp), Agios Pharmaceuticals, Pharmacosmos, and Roche.

## Supporting information


**Data S1** Supporting Information.
