# Supplementary material for: Non‐Transfusion‐Dependent Thalassemia: An Image Gallery Worth a Thousand Words
Source: Am J Hematol. 2025 Jan 30;100(4):687–94. doi: 10.1002/ajh.27621 (PMC11886484; doi:10.1002/ajh.27621)
Supplement: Supplementary file 1 — Data S1 Supporting Information. [file AJH-100-687-s001.pdf]

Non-transfusion-dependent thalassemia

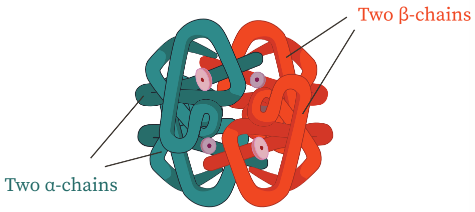

Thalassemia is an inherited disorder of hemoglobin, a molecule found in our red blood cells that is composed of four protein (globin) chains (2 α and 2 β), essential for oxygen delivery throughout the body.[1]

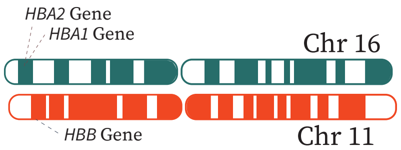

Patients who inherit genetic mutations affecting the α-globin chain have α-thalassemia and those with deficient β-globin chains have β-thalassemia.[1,2]

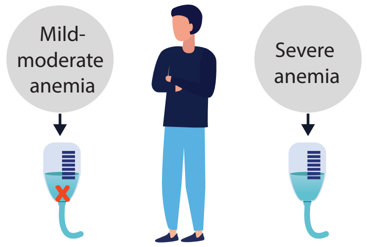

Thalassemia patients who were initially diagnosed with mild-moderate anemia and not placed on regular transfusion programs are known to have non-transfusion-dependent thalassemia (NTDT, which includes patients with β-thalassemia intermedia, mild/moderate hemoglobin E/β-thalassemia, and hemoglobin H disease).[3,4]

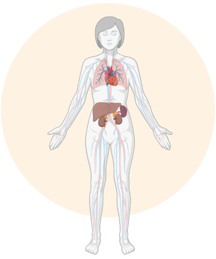

Recent studies have shown that, without treatment, patients with NTDT are at risk of various clinical complications and diminished quality of life, especially as they advance in age.[2,5,6]

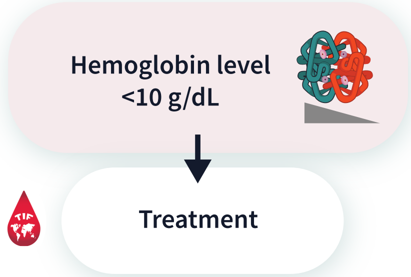

Specifically, patients with hemoglobin levels <10 g/dL are at an increased risk of complications and early death.[7-9] The Thalassaemia International Federation (TIF) now recommends treatment for patients with a hemoglobin level <10 g/dL, especially those who are symptomatic.[10]

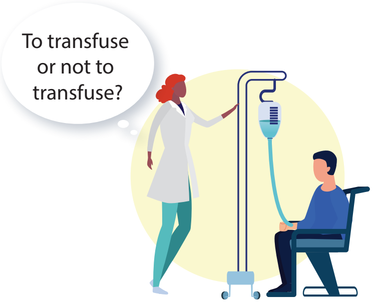

Until recently, transfusions were the only means to manage anemia in patients with NTDT. Although these may be effective, they increase the risk of iron overload and associated complications.[6,10,11]

BEYOND trial

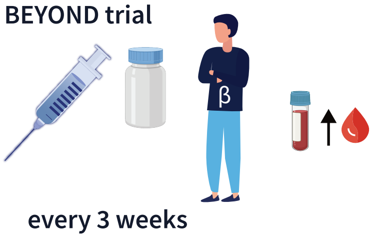

A clinical trial (BEYOND) has recently shown that **luspatercept,** a subcutaneous drug taken every three weeks, can improve hemoglobin level in adults with NTDT (β-thalassemia only), and the drug is now approved in Europe (but not the US).[12]

ENERGIZE trial

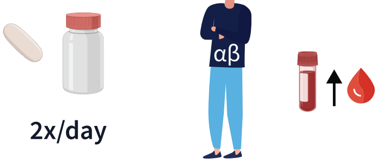

Mitapivat, which is taken orally twice per day, was also recently shown to improve hemoglobin level as well as functional status (fatigue) in adults with NTDT (both α- and β-thalassemia) in the ENERGIZE trial, and is now being reviewed for approval by regulatory agencies in Europe and the US.[13]

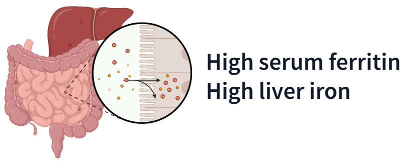

Iron overload can still occur in NTDT without transfusions, due to increased gut absorption.[14] Patients who have serum ferritin ≥800 ng/mL or liver iron concentration ≥5 mg/g have increased risks of complications and early death.[8,15-17] NTDT patients should be regularly monitored for iron overload and receive iron chelation if they reach these levels.[2,10,18,19]

NTDT patients should be regularly monitored for symptoms and signs of complications of the heart, blood vessels, liver, endocrine glands, bone, and other relevant body systems from adolescence or earlier if feasible. Tests should be performed more frequently with abnormal findings and patients should be referred to specialist care for management.[2,10,20]

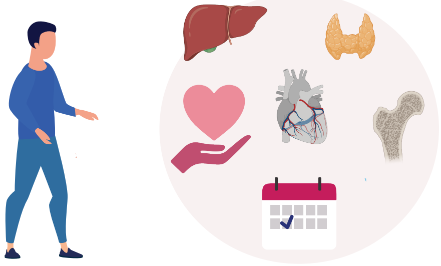

References

1. Taher AT, Musallam KM, Cappellini MD. beta-Thalassemias. *N Engl J Med.* 2021;384(8):727-743.
2. Musallam KM, Cappellini MD, Coates TD, et al. Alpha-thalassemia: A practical overview. *Blood Rev.* 2024;64:101165.
3. Musallam KM, Cappellini MD, Viprakasit V, Kattamis A, Rivella S, Taher AT. Revisiting the non-transfusion-dependent (NTDT) vs. transfusion-dependent (TDT) thalassemia classification 10 years later. *Am J Hematol.* 2021;96(2):E54-E56.
4. Musallam KM, Rivella S, Vichinsky E, Rachmilewitz EA. Non-transfusion-dependent thalassemias. *Haematologica.* 2013;98(6):833-844.
5. Taher AT, Musallam KM, El-Beshlawy A, et al. Age-related complications in treatment-naive patients with thalassaemia intermedia. *Br J Haematol.* 2010;150(4):486-489.
6. Taher AT, Musallam KM, Karimi M, et al. Overview on practices in thalassemia intermedia management aiming for lowering complication rates across a region of endemicity: the OPTIMAL CARE study. *Blood*. 2010;115(10):1886-1892.
7. Musallam KM, Cappellini MD, Daar S, Taher AT. Morbidity-free survival and hemoglobin level in non-transfusion-dependent beta-thalassemia: a 10-year cohort study. *Ann Hematol.* 2022;101(1):203-204.
8. Musallam KM, Vitrano A, Meloni A, et al. Risk of mortality from anemia and iron overload in nontransfusion-dependent beta-thalassemia. *Am J Hematol.* 2022;97(2):E78-E80.
9. Bizri M, Koleilat R, Akiki N, et al. Quality of life, mood disorders, and cognitive impairment in adults with beta-thalassemia. 2024;65:101181.
10. Taher A, Musallam K, Cappellini MD. *Guidelines for the Management of Non-Transfusion-Dependent* β-*Thalassaemia.* 3rd ed. Nicosia, Cyprus: Thalassaemia International Federation; 2023.
11. Musallam KM, Vitrano A, Meloni A, et al. Survival and causes of death in 2,033 patients with non-transfusion-dependent beta-thalassemia. *Haematologica*. 2021;106(9):2489-2492.
12. Taher AT, Cappellini MD, Kattamis A, et al. Luspatercept for the treatment of anaemia in non-transfusion-dependent beta-thalassaemia (BEYOND): a phase 2, randomised, double-blind, multicentre, placebo-controlled trial. *Lancet Haematol.* 2022;9(10):e733-e744.
13. Taher A, Al-Samkari H, Aydinok Y, et al. ENERGIZE: A Global Phase 3 Study of Mitapivat Demonstrating Efficacy and Safety in Adults with Alpha- or Beta- Non–Transfusion-Dependent Thalassemia [abstract]. *Hemasphere.* 2024;8(S1):11-12.
14. Rivella S. Iron metabolism under conditions of ineffective erythropoiesis in beta-thalassemia. Blood. 2019;133(1):51-58.
15. Musallam KM, Cappellini MD, Daar S, et al. Serum ferritin level and morbidity risk in transfusion-independent patients with beta-thalassemia intermedia: the ORIENT study. *Haematologica.* 2014;99(11):e218-221.
16. Musallam KM, Cappellini MD, Wood JC, et al. Elevated liver iron concentration is a marker of increased morbidity in patients with beta thalassemia intermedia. *Haematologica*. 2011;96(11):1605-1612.
17. Premawardhena AP, Ediriweera DS, Sabouhanian A, et al. Survival and complications in patients with haemoglobin E thalassaemia in Sri Lanka: a prospective, longitudinal cohort study. *Lancet Glob Health*. 2022;10(1):e134-e141.
18. Calvaruso G, Vitrano A, Di Maggio R, et al. Deferiprone versus deferoxamine in thalassemia intermedia: Results from a 5-year long-term Italian multicenter randomized clinical trial. *Am J Hematol.* 2015;90(7):634-638.
19. Taher AT, Porter J, Viprakasit V, et al. Deferasirox reduces iron overload significantly in nontransfusion-dependent thalassemia: 1-year results from a prospective, randomized, double-blind, placebo-controlled study. *Blood.* 2012;120(5):970-977.
20. Saliba AN, Musallam KM, Taher AT. How I treat non-transfusion-dependent beta-thalassemia. *Blood.* 2023;142(11):949-960.
